# Supplementary material for: A small area model to assess temporal trends and sub-national disparities in healthcare quality
Source: Nat Commun. 2023 Jul 28;14:4555. doi: 10.1038/s41467-023-40234-9 (PMC10382513; doi:10.1038/s41467-023-40234-9)
Supplement: Supplementary file 1 — Supplementary Information [file 41467_2023_40234_MOESM1_ESM.pdf]

Supplementary information file to "A small area model to assess temporal trends and sub-national disparities in healthcare quality using facility surveys"

This supplementary file provides supplemental data, figures, and more detailed results for "**A small area model to assess temporal trends and sub-national disparities in healthcare quality using facility surveys**"

Table of contents

List of figures and tables....2
Figures....2
Tables....2
Section 1. Supplementary Information....3
Section 1.1. Samples of the SDI and SPA surveys....3
Section 1.2. Geolocating health facilities....4
Section 1.3. Sampling of sick-child visits and vignettes....4
Section 1.4 Differences between the SPA and SDI surveys....4
Table S3. Covariate data sources....5
Section 2. Supplementary Methods....5
Section 2.1 List of tracer items and protocols included in the three countries, to estimate readiness and process quality of care....5
Section 2.2 Model for sub-national estimation of readiness and process quality of care metrics....7
Section 2.3 Spatial random effects....7
Section 2.4 Accounting for the sampling design in Senegal....8
Section 2.5 Model selection....8
Section 3. Supplementary Results....9
Section 3.1 Item availability by country....9
Section 3.2 Survey estimates of readiness and process quality of care metrics....11
Section 3.3 Model selection results....12
Section 3.4 Decomposition of the sources of variations in the readiness and process quality metrics....13
Section 3.5 Comparison of subnational-level survey and model estimates for the readiness and process quality metrics in Kenya and Tanzania....13
Section 3.6 Examples of model outputs in Kenya and Tanzania....20
Supplementary References....27

List of figures and tables

Figures

Supplementary Figure S1: Comparison of county-level survey and model estimates for the readiness and process quality metrics in Kenya, in 2018.
Supplementary Figure S2: Comparison of region-level survey and model estimates for the readiness and process quality metrics in Tanzania, in 2016.
Supplementary Figure S3: Regression coefficients from small area model that included covariates, across indicators.
Supplementary Figure S4: Measures of (Panel A) calibration, and (Panel B) bias and precision of models' predictions for stratified analysis, using hold-out predictions of readiness and process quality metrics in departments of Senegal.
Supplementary Figure S5: Maps of model-estimated readiness (panel A) and process quality (panel B) metrics by subnational areas in Kenya in 2020, with associated uncertainty
Supplementary Figure S6: Maps of model-estimated readiness (panel A) and process quality (panel B) metrics by subnational areas in Tanzania in 2020, with associated uncertainty
Supplementary Figure S7: Maps of model-estimated readiness (top panel) and process quality (bottom panel) metrics by subnational areas, and managing authorities, in Kenya in 2020
Supplementary Figure S8: Maps of model-estimated readiness (top panel) and process quality (bottom panel) metrics by subnational areas, and managing authorities, in Tanzania in 2020
Supplementary Figure S9: Maps of model-estimated readiness (left panel) and process quality (right panel) metrics by subnational areas, and managing authorities, in Kenya in 2020.

Tables

Table S1. Characteristics of health facilities sampled in the SPA surveys, in Senegal, Kenya, and Tanzania
Table S2. Characteristics of health facilities sampled in the SDI surveys, in Senegal, Kenya, and Tanzania
Table S3. Covariate data sources
Table S4. List of items used to derive the readiness metric, and differences between the three countries
Table S5. List of diagnostic protocols used to derive the process quality metric
Table S6. Models considered to estimate readiness and process quality metrics over time and space
Table S7. Items availability in Kenya for each round of SPA/SDI survey
Table S8. Items availability in Senegal for each round of SPA survey
Table S9. Items availability in Tanzania for each round of SPA/SDI survey
Table S10: Survey estimates of the national average and the range across subnational areas of the readiness and process quality metrics in Kenya, Senegal, and Tanzania
Table S11: Models' fit assessed using WAIC, DIC, and LCPO
Table S12. Selected models and variance decomposition, for each readiness and process quality of care metrics in each country. $\sigma^2_{S_i}$, $\sigma^2_{\gamma_t}$, $\sigma^2_{\alpha_t}$, $\sigma^2_{\delta_{it}}$, $\sigma^2_{\upsilon_t}$, and $X_{it}\beta$ columns indicate the percentage of total variance explained by each component.

Section 1. Supplementary Information

Section 1.1. Samples of the SDI and SPA surveys

In Senegal, data were collected as part of a Continuous SPA, which achieved a census of all facilities after five rounds of surveys. Half of the hospitals and health centers were selected in the first (2012-13) and third round (2015) of SPA, while the other half were selected in the second (2014) and fourth round (2016), which created a dependent sampling structure between the first four rounds of the continuous SPA. Random samples of facilities were selected for the SPA conducted in 2017, 2018 and 2019. As the higher levels in the process quality metrics, observed in years 2015 and 2016 compared to 2012-13 and 2014, were not found in the later years, we can hypothesize that the facilities and providers who were observed for the second time in 2015 and 2016, had a better knowledge of SPA interviewers' assessment criteria, which could have led to an enhanced Hawthorne effect.

	**Kenya**			**Tanzania**	
	SPA 1999	*SPA 2004*	*SPA 2010*	*SPA 2006*	*SPA 2014-15*
**Facility type**					
Hospital	32	172	253	128	263
Health center	90	51	101	41	380
Clinic	256	217	349	437	557
**Managing authority**					
Public	177	175	351	425	783
Private	211	265	352	186	417
**Total**	388	440	703	611	1200

			**Senegal**		
	SPA 2013/14	*SPA 2015/16*	*SPA 2017*	*SPA 2018*	*SPA 2019*
**Facility type**					
Hospital	70	73	35	29	31
Health center	126	126	74	62	64
Clinic	531	557	287	248	246
**Managing authority**					
Public	598	587	315	270	288
Private	129	159	81	69	73
**Total**	727	746	396	339	361

Table S1: Characteristics of health facilities sampled in the SPA surveys, in Senegal, Kenya, and Tanzania

	Tanzania			Kenya		Senegal
	SDI 2010	*SDI 2014*	*SDI 2016*	*SDI 2012*	*SDI 2018*	*SDI 2010*
**Facility type**						
Hospital	Not reported	27	30	51	161	0
Health center	Not reported	84	92	62	484	111
Clinic	Not reported	272	264	100	2,449	41
**Managing authority**						
Public	175	269	273	158	1,781	151
Private	0	134	127	134	1,313	0
**Total**	175	383	386	292	3,094	151

Table S2: Characteristics of health facilities sampled in the SDI surveys, in Senegal, Kenya, and Tanzania

Section 1.2. Geolocating health facilities

For SPA survey data, we determined in which regions each facility was located using the GPS coordinates provided by the DHS program and the second-level administrative shapefiles that are publicly available from the Database of Global Administrative Areas (https://gadm.org/). The two most recent SPA surveys in Senegal did not collect GPS coordinates, but DHS provided a linkage file to assign a department to each sampled facility. SDI surveys include the administrative units as a variable directly.

Section 1.3. Sampling of sick-child visits and vignettes

In the SPA, the survey teams randomly selected three providers of curative care of sick children among all providers in this service present at the facility the day of the assessment. A maximum of five client consultations for each selected provider was observed. The client weights use the facility sampling weight as its base weight and take into account the total number of clients listed and interviewed within each of the sampling stratum, to calculate the probability of a given consultation to be observed. In the SDI, vignettes were administered to a random sample of health providers (doctors, medical assistants, nurses) among all eligible providers present at the facility the day of the assessment. Providers were randomly sampled among all eligible providers at the facility. The weights here simply represent the percentage probability of selection of a provider within each facility.

Section 1.4 Differences between the SPA and SDI surveys

The SPA and SDI surveys' inventory questionnaire mostly collect the same information, as both surveys use WHO's SARA framework to assess the availability and readiness of key infrastructure and services in facilities, what we referred to as readiness in this study.

However, the differences in the methodology used to assess providers' knowledge and competence, referred to as process quality in this article, differs significantly. In the SPA survey, interviewers directly observe patient-provider consultations using an observation protocol. In the SDI survey, interviewers act as patients and record providers' questions, examinations, and recommendations to a hypothetical clinical case, known as vignette. Past studies have shown that compliance to protocol checklists tend to be higher with vignettes than with direct clinical observations. To account for these differences in the assessment of process quality between the SPA and SDI surveys, we used survey-specific effects in our model.

Covariate	Spatial resolution	Temporal resolution	Source
Total population under five years old	Pixel-level	Annual	WorldPop
Travel time to nearest settlement >50,000 inhabitants	Pixel-level	Annual	WorldPop
Travel time to nearest health facility	Pixel-level	Annual	WorldPop
Health worker density	Administrative unit	Census years	Censuses (derived)
Urbanicity	Pixel-level	2015	ESA and land cover
Night-time lights	Pixel-level	Annual	VIIRS and DMSP (harmonized)
Educational attainment	Pixel-level	Annual	IHME
Human development index	Pixel-level	Annual	IHME
Elevation	Pixel-level	Annual	ArcGIS

DMSP=Defense Meteorological Satellite Program; ESA=European Space Agency; IHME=Institute for Health Metrics and Evaluation; VIIRS= Visible Infrared Imaging Radiometer Suite

Table S3. Covariate data sources

Section 2. Supplementary Methods

Section 2.1 List of tracer items and protocols included in the three countries, to estimate readiness and process quality of care

	Included (X)

	Kenya	Senegal	Tanzania
**Readiness**			
Electricity	X	X	
Improved water	X	X	
Privacy of examination room		X	
Improved latrine for client use	X	X	
Communication equipment	X	X	X
Computer with email	X	X	X
Emergency transport	X	X	
Disposal of sharps	X	X	X
Disposal of medical waste	X	X	X
Monthly administrative meetings		X	
Quality assurance system		X	
System to collect opinion		X	
Supervision in the last six months		X	
Health workers always available		X	
Guidelines for IMCI	X	X	X
Child scale	X	X	X
Infant scale	X	X	X
Thermometer	X	X	X
Stethoscope	X	X	X
Amoxicillin for children	X	X	X
ORS	X	X	X
Co-trimoxazole for children	X	X	X
Paracetamol	X	X	X
Malaria diagnostic capacity	X	X	X
Antimalarial medication	X	X	X

Table S4: List of tracer items and diagnostic protocols used to derive the readiness metric, and differences between the three countries

	Protocols
**Process quality of care**	
History taking	Provider asked about cough or difficulty breathing, diarrhoea, fever, inability to drink anything, vomiting, and convulsions

Physical examination	Provider took child's temperature, checked for pallor, looked in child's ear, counted respiration, checked skin turgor for dehydration, undressed child to examine, weighed the child, pressed both feet to check for oedema

Table S5: List of diagnostic protocols used to derive the process quality metric

Section 2.2 Model for sub-national estimation of readiness and process quality of care metrics

The two composite indices of readiness and process quality of care described in table 2 were modelled separately using a small area estimation approach. Specifically, we adapted a previously developed Bayesian framework, which models the direct survey estimates as a function of covariates, and space and time components [1]. This Bayesian spatial model allows to estimate time series of the true underlying values of the metrics by smoothing over time and space the direct estimates obtained from multiple surveys, with potentially different designs and associated uncertainty. Specifically, in the first stage, we calculate the Horvitz-Thompson estimator of the metric in area *i*, year *t*, and survey *s*, by using the sampling weight *w* associated to each facility *k*:

$p_{its}{}^{HT} = \frac{\sum_{k \in \mathrm{i}} w_{k,its} y_{k,its}}{\sum_{k \in \mathrm{i}} w_{k,its}}$ with variance V_i^*calculated using standard methods, such as jackknife.

To increase the precision of the design-based estimates of the metric, and to predict the metric in areas or years where no data was collected, we use a hierarchical spatial model inspired by Fay and Herriot classic framework:

$$\mathrm{logit}(p_{its}{}^{HT}) \sim N(\theta_{its}, V_{its})$$

$$\theta_{its} = \mathbf{X}_{\mathrm{it}}\boldsymbol{\beta} + \gamma_t + \alpha_t + e_i + S_i + \delta_{it} + \upsilon_s$$

Where $\mathbf{X}_{\mathrm{it}}\boldsymbol{\beta}$ are area-level covariates for area *i* in year *t* (see Table S3), γ_t are temporal random effects modeled as a first order random walk, S_i are spatially structured random effects (see section 2.3), δ_{it} is a space-time interaction, α_t, e_i and υ_s are temporal, spatial and survey zero-mean independent random effects.

Section 2.3 Spatial random effects

We model area-level random effects using an intrinsically conditional autoregressive model known as the BYM2 model- an extension of the BYM model originally developed by Besag, York, and Mollie[2], which adds penalized priors[3]. The use of spatially structured random effects reflects our assumption that these unobserved characteristics affecting the availability of quality care are likely to be correlated in space. For instance, remote administrative units might face challenges - in accessing steady drug supply chains for essential medicines and testing materials, or in attracting qualified health workers - that are more like adjacent units than that experienced in the capital city. Models 1-4 are alternative where area-level random effects are assumed to be independent and identically distributed rather than spatially correlated.

Section 2.4 Accounting for the sampling design in Senegal

In Senegal, to account for the changing sampling methods (described in B.1.1), we adopted an analytical approach that includes random effects to account for the rounds of SPA that comprised repeated selection of facilities.

Section 2.5 Model selection

Direct estimates and design-based variance estimates were computed using the ***survey*** package in R version 4.0.1. We fit the Bayesian hierarchical models using the Integrated Nested Laplace Approximation [4] and the ***R-INLA*** package [5] version 22.12.16. We obtained a subset of all included covariates by checking for multi-collinearity using the variance inflation factor with a threshold of 5. For each indicator and country, we compared the 7 models presented in Table S4 consisting of different combinations of the covariates selected with the variance inflation factor (VIF) procedure[6], and spatio-temporal random effects, using three selection procedures (the deviance information criteria[7], the Watanabe-Akaike information criteria [8], and the sum of log-conditional predictive ordinate [9]). In the absence of consensus on a single criterion, when different criteria pointed to different models, we used a majority rule.

Model	Formula
1	$\gamma_t + \alpha_t + e_i + \delta_{it}$
2	$\gamma_t + \alpha_t + e_i + \delta_{it} + \upsilon_s$
3	$\mathbf{X_{it}\beta} + \gamma_t + \alpha_t + e_i + \delta_{it}$
4	$\mathbf{X_{it}\beta} + \gamma_t + \alpha_t + \delta_{it} + e_i + \upsilon_s$
5	$\gamma_t + \alpha_t + S_i + e_i + \delta_{it}$
6	$\gamma_t + \alpha_t + S_i + \delta_{it} + e_i + \upsilon_s$
7	$\mathbf{X_{it}\beta} + \gamma_t + \alpha_t + S_i + e_i + \delta_{it} + \upsilon_s$

Table S6: Models considered to estimate readiness and process quality metrics over time and space

Section 3. Supplementary Results

Section 3.1 Item availability by country

**Kenya**	**1999**	**2004**	**2010**	**2018**
Electricity	58%	47%	26%	56%
Improved water	80%	85%	83%	97%
Privacy of examination room	78%	76%	83%	100%
Improved latrine for client use	98%	97%	97%	100%
Communication equipment	63%	47%	82%	75%
Computer/internet/email	---	8%	14%	74%
Emergency transport	26%	20%	10%	75%
Disposal of sharps	47%	75%	64%	97%
Disposal of medical waste	47%	97%	88%	---
Monthly administrative meeting	51%	61%	49%	30%
Quality assurance	30%	24%	23%	28%
Systematic collection of clients' opinion	47%	61%	59%	32%
Supervision in last 6 Months	56%	87%	83%	---
Health workers available at all time	63%	47%	37%	77%
IMCI guidelines available	61%	11%	34%	10%
Child scale	85%	60%	68%	58%
Infant scale	85%	73%	68%	52%
Thermometer	86%	81%	88%	94%
Stethoscope	100%	78%	83%	97%
Amoxicillin	65%	78%	60%	79%
ORS	86%	88%	75%	80%
Co-trimoxazole	86%	90%	78%	85%
Paracetamol	80%	88%	69%	92%
Malaria diagnostic tools	---	50%	45%	53%
Antimalarial medication	37%	80%	94%	83%
**Unweighted mean**	**66%**	**64%**	**63%**	**70%**

Table S7: Items availability in Kenya for each round of SPA/SDI survey. The unweighted mean differs from the readiness metric presented in Table 3, as items are grouped and averaged by domains (infrastructure, facility management, drug availability) before being aggregated as the final readiness metric.

Senegal	2013/14	2015/16	2017	2018	2019
Electricity	54%	55%	61%	56%	74%
Improved water	92%	94%	94%	97%	97%
Privacy of examination room	99%	97%	99%	100%	96%
Improved latrine for client use	91%	94%	99%	100%	96%
Communication equipment	54%	45%	55%	75%	85%
Computer/internet/email	53%	42%	61%	74%	78%
Emergency transport	51%	57%	57%	75%	63%
Disposal of sharps	87%	96%	92%	97%	88%
Disposal of medical waste	---	---	---	---	---
Monthly administrative meeting	29%	36%	43%	30%	49%
Quality assurance	4%	9%	31%	28%	28%
Systematic collection of clients' opinion	11%	14%	27%	32%	43%
Supervision in last 6 Months	---	---	---	---	---
Health workers available at all time	85%	85%	84%	77%	71%
IMCI guidelines available	46%	64%	75%	58%	65%
Child scale	73%	76%	52%	49%	52%
Infant scale	70%	83%	86%	75%	71%
Thermometer	98%	99%	98%	99%	95%
Stethoscope	99%	100%	98%	99%	97%
Amoxicillin	82%	81%	78%	70%	68%
ORS	78%	74%	76%	68%	64%
Co-trimoxazole	71%	58%	17%	29%	30%
Paracetamol	90%	89%	88%	84%	75%
Malaria diagnostic tools	75%	90%	85%	82%	76%
Antimalarial medication	61%	47%	64%	59%	54%
**Unweighted mean**	**71%**	**71%**	**73%**	**73%**	**72%**

Table S8: Items availability in Senegal for each round of SPA survey. The unweighted mean differs from the readiness metric presented in Table 3, as items are grouped and averaged by domains (infrastructure, facility management, drug availability) before being aggregated as the final readiness metric.

Tanzania	2006	2014	2014-15	2016
Communication equipment	45%	30%	51%	40%
Computer/internet/email	4%	9%	12%	12%
Disposal of sharps	83%	87%	34%	81%
Disposal of medical waste	97%	88%	36%	82%
IMCI guidelines available	34%	58%	54%	80%
Child scale	76%	83%	79%	78%
Infant scale	54%	64%	72%	82%
Thermometer	87%	91%	85%	93%
ORS	84%	76%	85%	88%
Co-trimoxazole	81%	77%	86%	74%
Paracetamol	84%	76%	69%	74%
Malaria diagnostic tools	41%	81%	43%	94%
Antimalarial medication	99%	91%	94%	96%
**Unweighted mean**	**67%**	**71%**	**62%**	**73%**

Table S9: Items availability in Tanzania for each round of SPA/SDI survey. The unweighted mean differs from the readiness metric presented in Table 3, as items are grouped and averaged by domains (infrastructure, facility management, drug availability) before being aggregated as the final readiness metric.

Section 3.2 Survey estimates of readiness and process quality of care metrics

	Readiness		Process quality	
**Country and survey year**	**Mean survey estimate (95% CI)**	**Absolute range**	**Mean survey estimate (95% CI)**	**Absolute range**
**Kenya**				
SPA 1999	63.1 (61.7-64.5)	56.9-73.9	30.7 (29.7-31.7)	20.5-39.2
SPA 2004	65.6 (63.3-67.9)	59.4-72.7	35.5 (34.2-36.8)	23.3-74.0
SPA 2010	60.6 (59.2-62.0)	36.4-92.0	44.3 (43.3-45.5)	19.3-86.7
SDI 2018	65.1 (64.6-65.6)	52.4-83.3	43.7 (42.7-44.7)	26.9-73.5
**Senegal**				
SPA 2012-14	65.1 (64.3-65.9)	40.0-77.1	38.5 (37.8-39.2)	15.4-59.3
SPA 2015-16	65.7 (64.5-66.9)	37.1-82.9	43.1 (42.1-44.1)	19.2-63.7
SPA 2017	66.9 (65.5-68.3)	31.3-82.9	34.5 (33.5-35.5)	19.5-54.4
SPA 2018	65.0 (59.6-70.4)	22.9-85.7	36.3 (32.5-40.1)	23.8-66.7
SPA 2019	64.6 (61.3-67.9)	31.4-85.7	39.0 (37.0-41.0)	7.7-67.2
**Tanzania**				
SPA 2006	54.4 (53.6-55.3)	42.2-68.8	39.8 (38.9-40.7)	10.4-50.3
SPA 2014-15	62.5 (61.4-63.6)	48.8-75.8	35.3 (34.7-36.0)	21.4-67.7
SDI 2014	73.0 (71.8-75.2)	58.2-92.2	36.4 (34.6-38.2)	25.5-54.1
SDI 2016	74.6 (72.6-76.6)	58.7-93.7	39.9 (37.1-42.7)	22.4-71.9

Table S10: Survey estimates of the national average and the range across subnational areas of the readiness and process quality metrics in Kenya, Senegal, and Tanzania. Estimates presented in this table are empirical survey estimates (as opposed to the modeled estimates presented in what follows). They were calculated separately for each SPA and SDI survey, using their respective survey weights and design variables. The absolute range for each survey was derived by estimating readiness and process quality by county (Kenya), department (Senegal), and region (Tanzania). Although these surveys were typically powered to provide reliable estimates at a less granular resolution (province, region, and zone, respectively), we present the absolute range to illustrate the width of subnational inequities within each country.

Section 3.3 Model selection results

Model	Readiness of care			Process quality of care		
	WAIC	*DIC*	*LCPO*	*WAIC*	*DIC*	*LCPO*
**Kenya**						
**1**	-239.68	121.82	54.97	-211.41	170.79	-91.44
**2**	**-239.71**	**121.84**	**55.02**	-211.47	170.80	-91.25
**3**	-238.03	122.57	52.68	-212.92	170.36	-89.43
**4**	-237.88	122.69	52.74	-212.79	170.49	-89.47
**5**	-239.88	120.99	58.00	-212.67	169.89	-84.13
**6**	-239.92	120.95	58.00	-212.58	169.95	-84.41
**7**	-238.43	122.00	**55.45**	**-214.31**	**169.93**	**-82.43**
**Senegal**						
**1**	-66.73	157.56	76.48	-147.75	180.47	85.15
**2**	-66.97	157.28	76.55	-147.78	180.33	85.07
**3**	-67.84	158.78	76.66	-147.29	181.14	87.02
**4**	-67.73	158.80	76.84	-147.04	181.20	87.04
**5**	**-66.94**	**157.34**	**75.61**	**-147.80**	**180.64**	**85.49**
**6**	-67.02	157.54	75.70	-147.67	180.61	85.56
**7**	-67.79	158.87	76.61	-147.23	181.09	87.32
**Tanzania**						
**1**	**-33.59**	**53.46**	**25.71**	-71.01	71.20	61.07
**2**	-33.27	53.37	25.43	-71.08	71.10	61.26
**3**	-31.90	53.54	25.57	**-71.68**	**71.42**	**60.04**
**4**	-31.90	53.54	25.57	-71.68	71.42	60.04
**5**	-32.95	53.45	26.29	-70.99	71.22	61.58
**6**	-33.15	53.26	26.12	-70.94	71.26	61.71
**7**	-31.47	53.56	25.97	-71.41	71.50	61.13

Table S11: Models' fit assessed using WAIC, DIC, and LCPO. Lower WAIC and DIC indicate better model performance, while higher LCPO indicates better model performance.

Section 3.4 Decomposition of the sources of variations in the readiness and process quality metrics

	Senegal							
Outcome	Model selected	$\sigma^2_{e_i}$	$\sigma^2_{S_i}$	$\sigma^2_{\gamma_t}$	$\sigma^2_{\alpha_t}$	$\sigma^2_{\delta_{it}}$	$\sigma^2_{\upsilon_s}$	$\mathbf{X_{it}\beta}$
Readiness of care	5	---	34.8	2.6	1.4	61.1	---	---
Process quality of care	5	---	0.6	1.9	2.2	95.3	---	---
	**Kenya**							
Outcome	Model selected	$\sigma^2_{e_i}$	$\sigma^2_{S_i}$	$\sigma^2_{\gamma_t}$	$\sigma^2_{\alpha_t}$	$\sigma^2_{\delta_{it}}$	$\sigma^2_{\upsilon_s}$	$\mathbf{X_{it}\beta}$
Readiness of care	2	6.7	---	10.3	24.6	1.5	56.9	---
Process quality of care	7	---	3.6	1.9	3.5	24.6	7.7	58.8
	**Tanzania**							
Outcome	Model selected	$\sigma^2_{e_i}$	$\sigma^2_{S_i}$	$\sigma^2_{\gamma_t}$	$\sigma^2_{\alpha_t}$	$\sigma^2_{\delta_{it}}$	$\sigma^2_{\upsilon_s}$	$\mathbf{X_{it}\beta}$
Readiness of care	3	0.6	---	6.3	57.3	12	---	23.8
Process quality of care	3	0.5	---	1.3	0.8	53.8	---	43.5

Table S12: Selected models and variance decomposition, for each readiness and process quality of care metrics in each country. The $\sigma^2_{e_i}, \sigma^2_{S_i}, \sigma^2_{\gamma_t}, \sigma^2_{\alpha_t}, \sigma^2_{\delta_{it}}, \sigma^2_{\upsilon_t}$, and $X_{it}\boldsymbol{\beta}$ columns indicate the percentage of total variance explained by the zero-mean spatial component, the spatially-structured component, the temporally-structured component, the zero-mean temporal component, the space-time interaction component, and the covariate component.

Section 3.5 Comparison of subnational-level survey and model estimates for the readiness and process quality metrics in Kenya and Tanzania

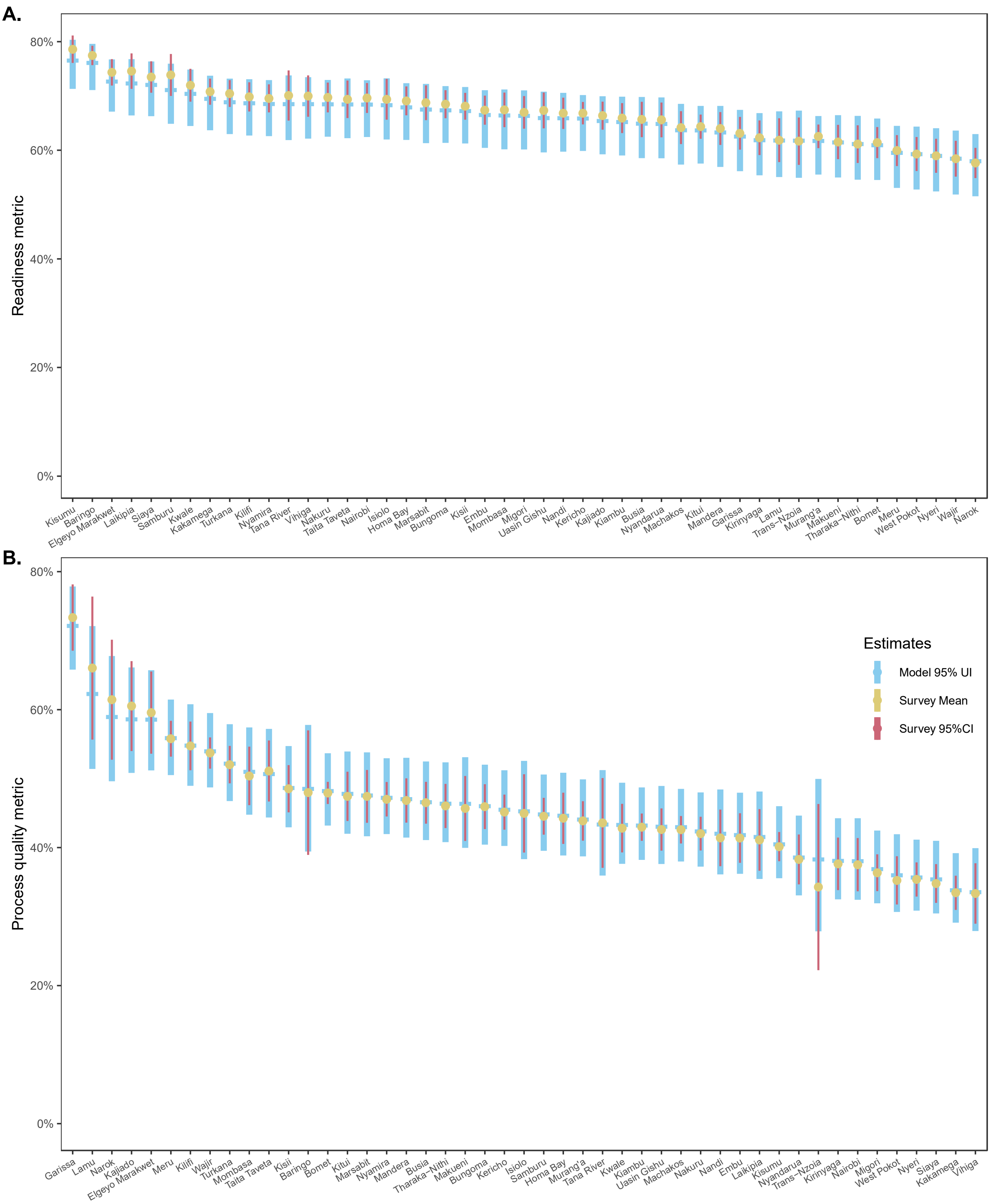

Supplementary Figure S1: Comparison of county-level survey and model estimates for the readiness and process quality metrics in Kenya, in 2018. This figure compares empirical

survey and model estimates, for the most recent year when data was available in Kenya. Thick light-blue dash and vertical ranges show model posterior mean estimates, and the 95% posterior prediction intervals. Yellow dots and narrow red vertical lines indicate survey estimates and 95% confidence intervals, derived from SDI 2018.

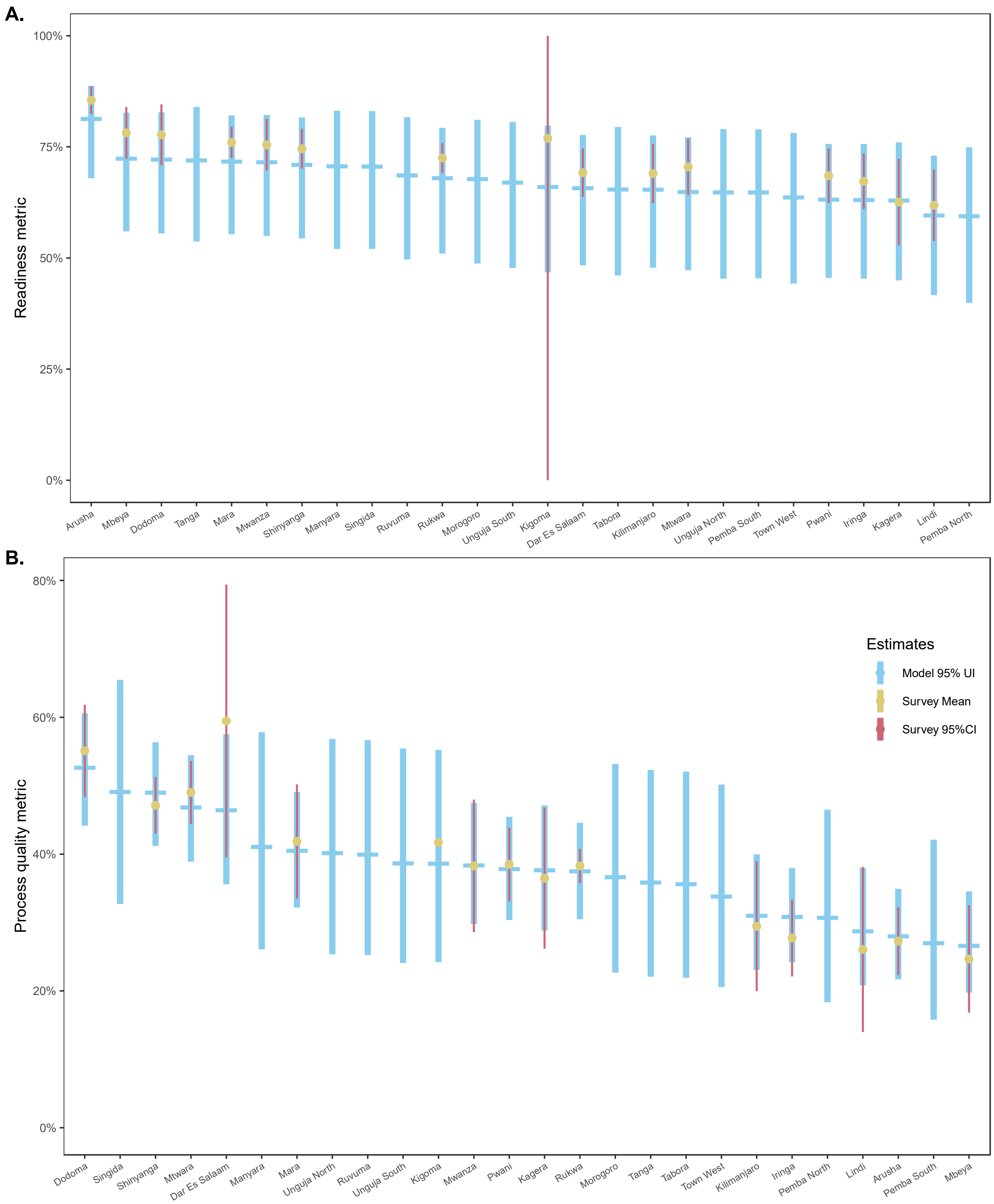

Supplementary Figure S2: Comparison of region-level survey and model estimates for the readiness and process quality metrics in Tanzania, in 2016. This figure compares

empirical survey and model estimates, for the most recent year when data was available in Tanzania. Thick light-blue dash and vertical ranges show model posterior mean estimates, and the 95% posterior prediction intervals. Yellow dots and narrow red vertical lines indicate survey estimates and 95% confidence intervals, derived from SDI 2016 (n = 386 facilities sampled, panel A; n = 543 providers assessed in 397 facilities, panel B).

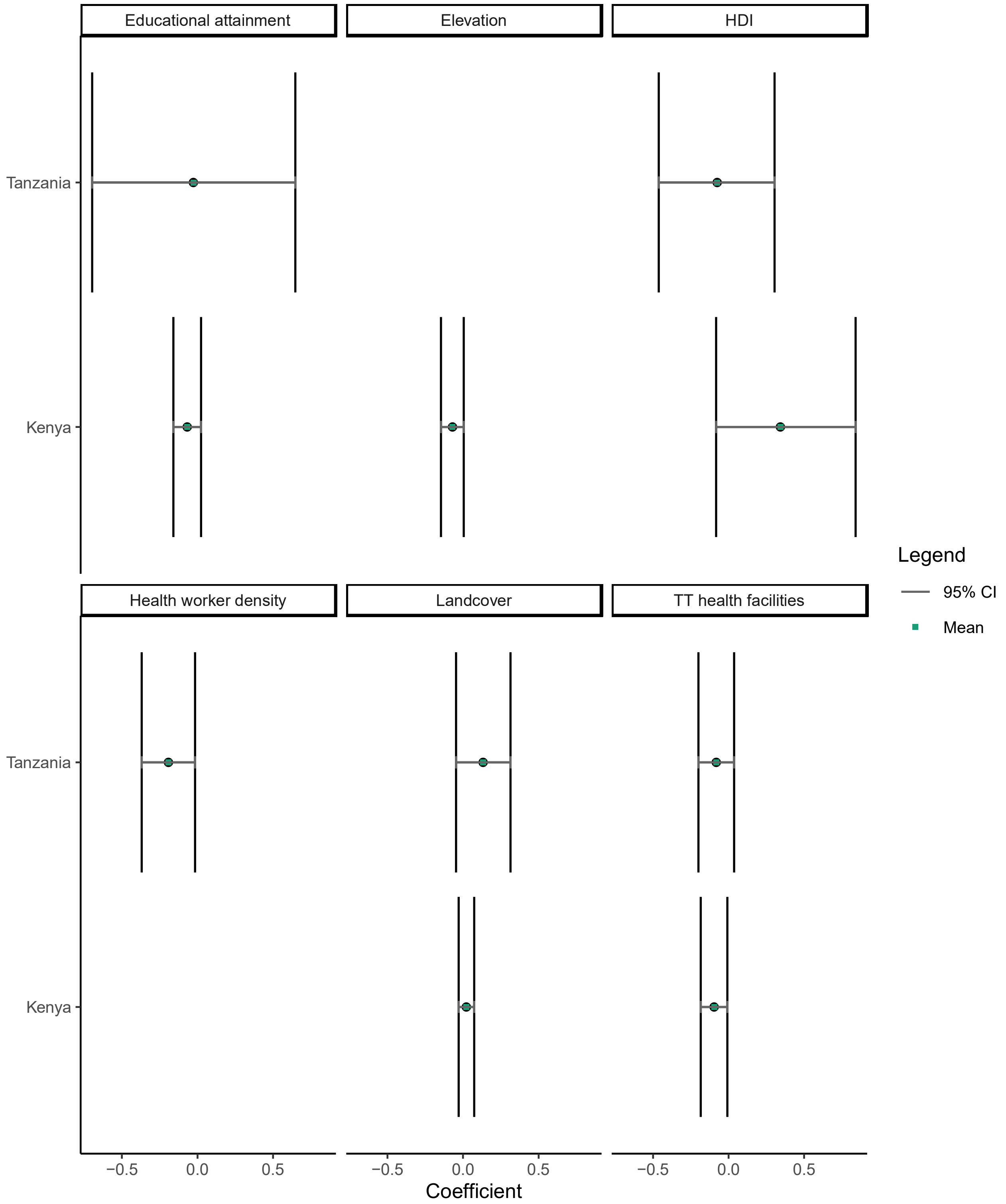

Supplementary Figure S3: Regression coefficients from small area models that included covariates (n = 1,974, Kenya; n = 884, Tanzania). All the variables were scaled for computational purposes.

Access = average travel time to nearest urban settlements with over 50,000 inhabitants; HDI = human development index; TT health facilities = travel time to nearest facility; Landcover = measure of urbanicity of the area; Elevation = average elevation of the area.

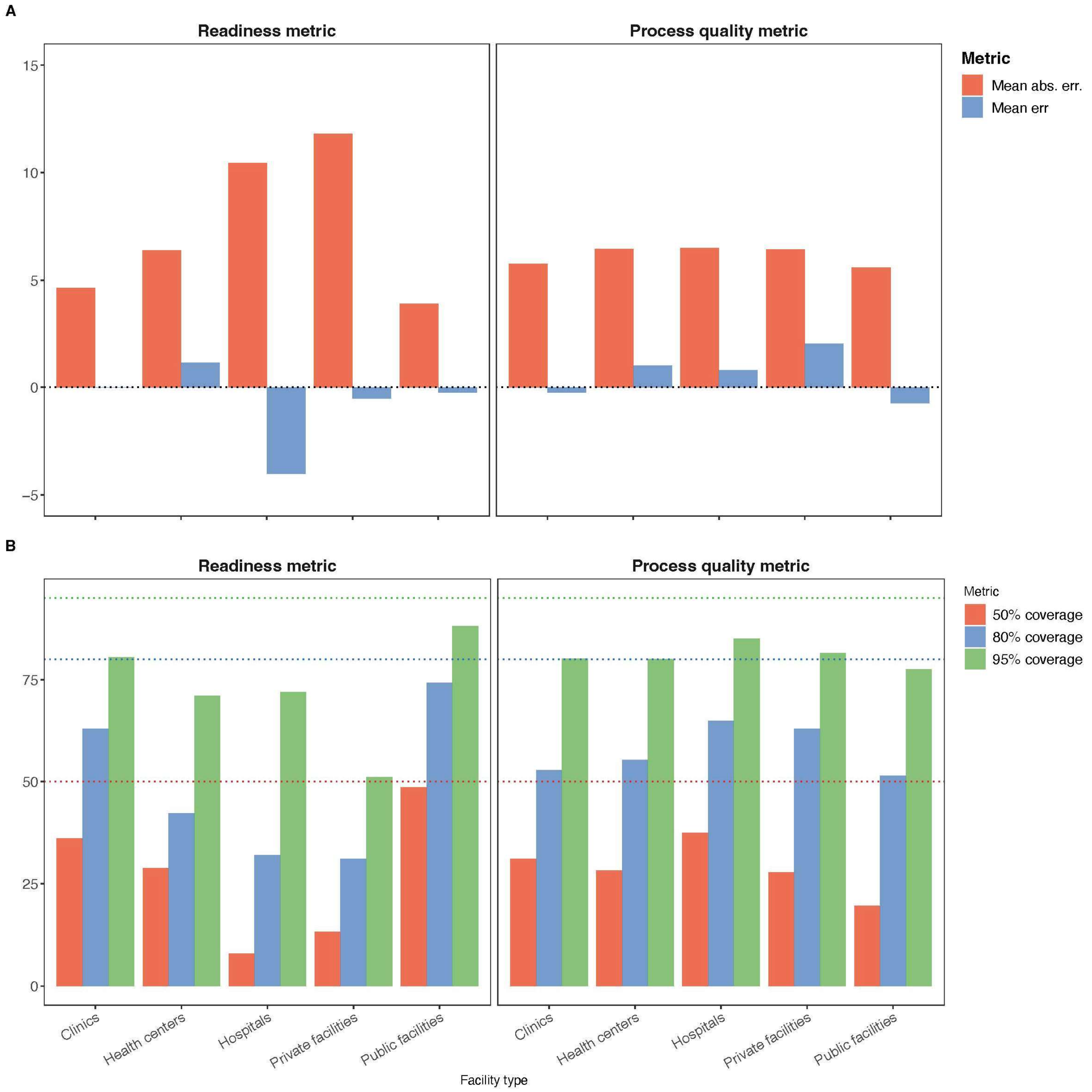

Supplementary Figure S4: Measures of (Panel A) calibration, and (Panel B) bias and precision of models' predictions for stratified analysis, using hold-out predictions of readiness and process quality metrics in departments of Senegal. Mean error, mean absolute error, and coverage were calculated across all administrative areas, using cross validation.

Stratified analyses only include one of five type of facilities- public facilities, private facilities, hospitals, health centers, and clinics.

Section 3.6 Examples of model outputs in Kenya and Tanzania

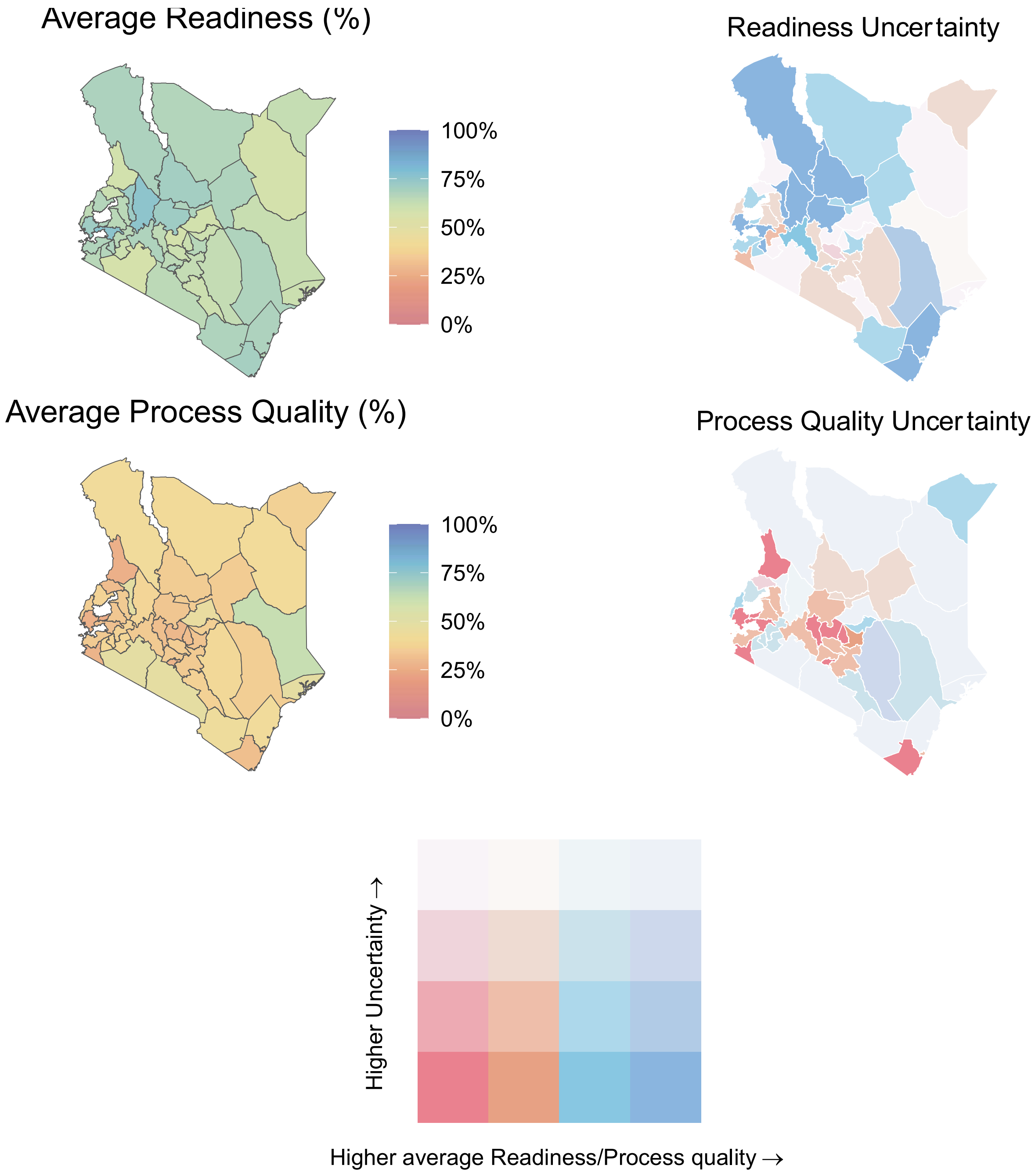

Supplementary Figure S5: Maps of model-estimated readiness (panel A) and process quality (panel B) metrics by subnational areas in Kenya in 2020, with associated uncertainty. The left panel presents estimates of the mean, while the right panel shows both estimates of the mean and their associated 95% uncertainty interval width. Mean estimated metrics are split into quartiles; the cut-off points indicate the metric estimates' minimum, 25th, 50th, and 75th percentiles, and maximum, which were 57.1%, 61.4%, 65.5%, 67.7%, and 75.8%, for the readiness metric, and 25.4%, 31.7%, 35.5%, 39.7%, and 61.9%, for process quality. The confidence intervals' width

minimum, 25th, 50th, and 75th percentiles, and maximum, were 21.0%, 24.6%, 25.4%, 26.4%, and 27.3%.

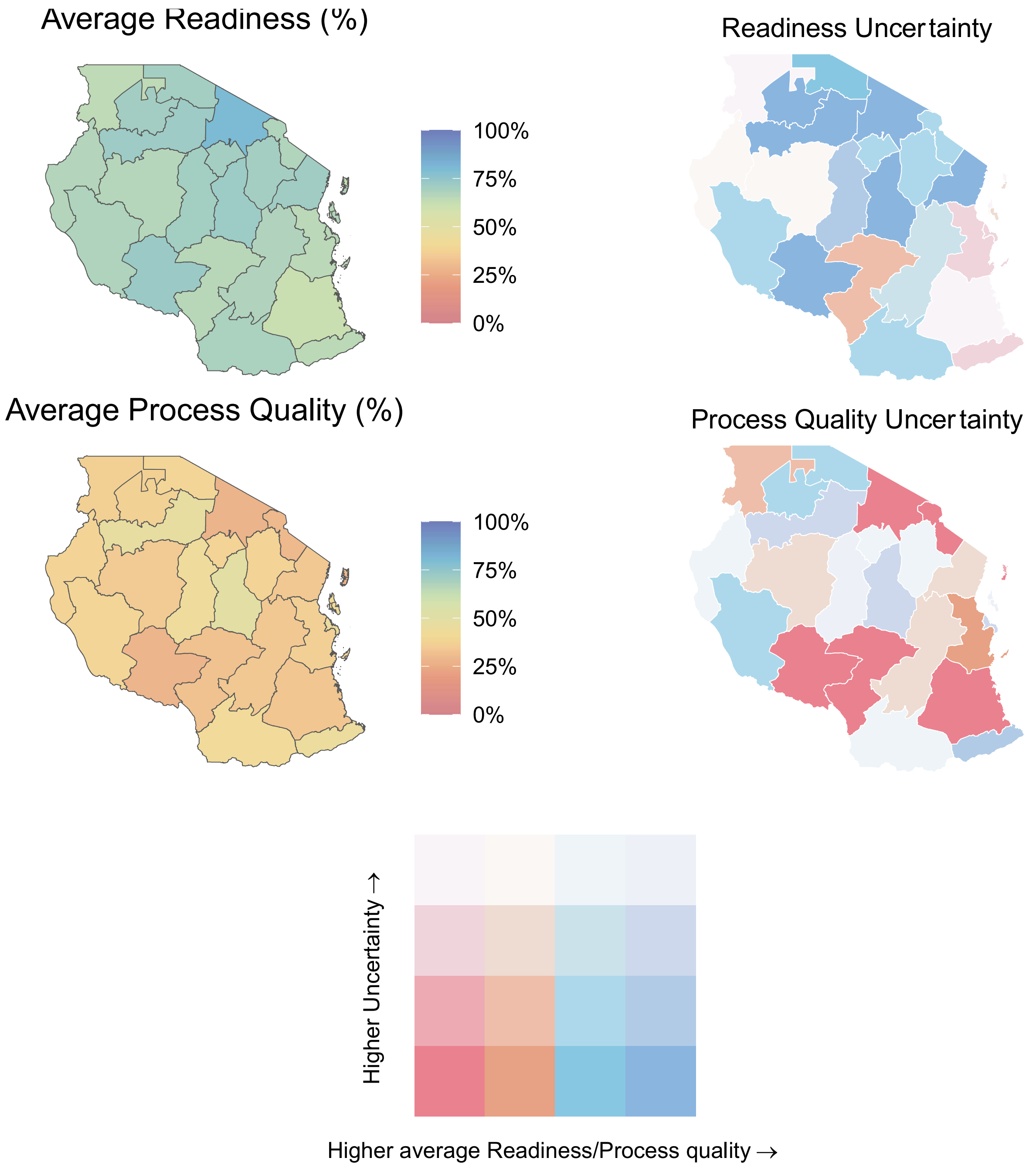

Supplementary Figure S6: Maps of model-estimated readiness (panel A) and process quality (panel B) metrics by subnational areas in Tanzania in 2020, with associated uncertainty. The left panel presents estimates of the mean, while the right panel shows both estimates of the mean and their associated 95% uncertainty interval width. Mean estimated metrics are split into

quartiles; the cut-off points indicate the metric estimates' minimum, 25th, 50th, and 75th percentiles, and maximum, which were 61.3%, 65.4%, 66.9%, 70.4%, and 80.0%, for the readiness metric, and 27.3%, 33.7%, 36.8%, 41.4%, and 49.5%, for process quality. The confidence intervals' width minimum, 25th, 50th, and 75th percentiles, and maximum, were 40.3%, 50.2%, 52.2%, 53.0%, and 55.0%.

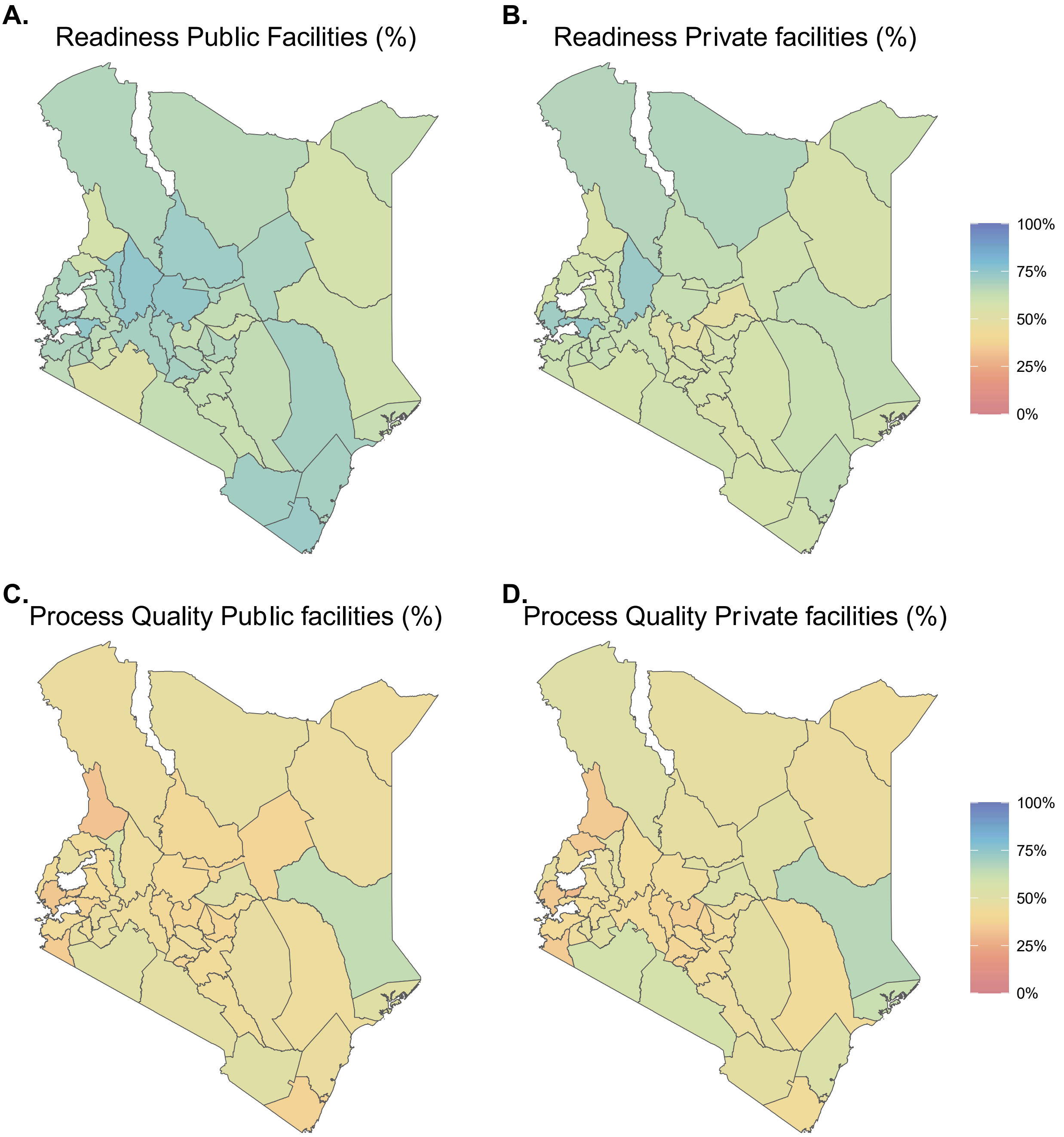

Supplementary Figure S7: Maps of model-estimated readiness (top panel) and process quality (bottom panel) metrics by subnational areas, and managing authorities, in Kenya

in 2020. Figures A and B (respectively C and D) are maps of modelled area-level estimates of readiness (respectively process quality) for analyses stratified on public and private facilities.

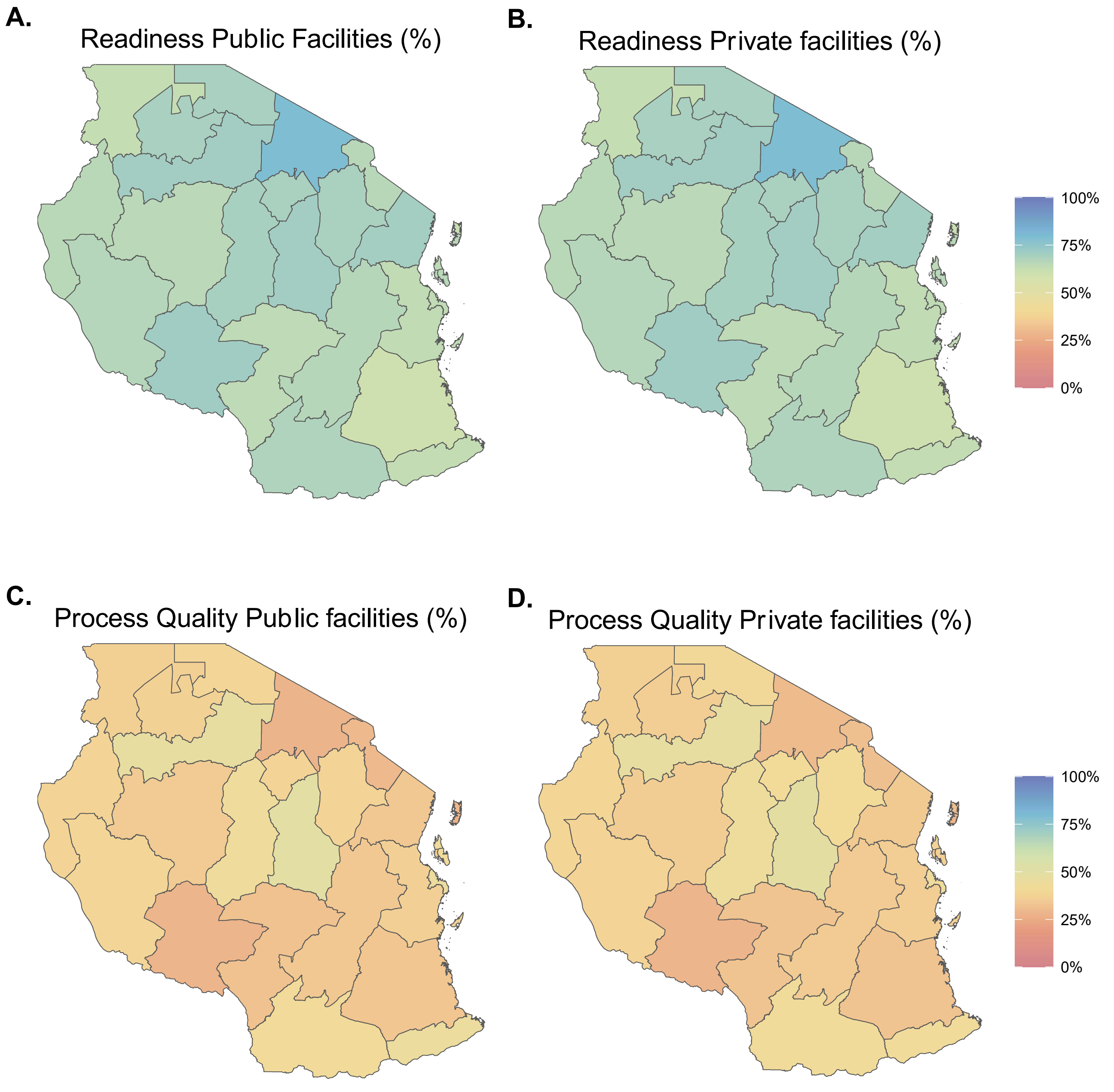

Supplementary Figure S8: Maps of model-estimated readiness (top panel) and process quality (bottom panel) metrics by subnational areas, and managing authorities, in Tanzania in 2020. Figures A and B (respectively C and D) are maps of modelled area-level estimates of readiness (respectively process quality) for analyses stratified on public and private facilities.

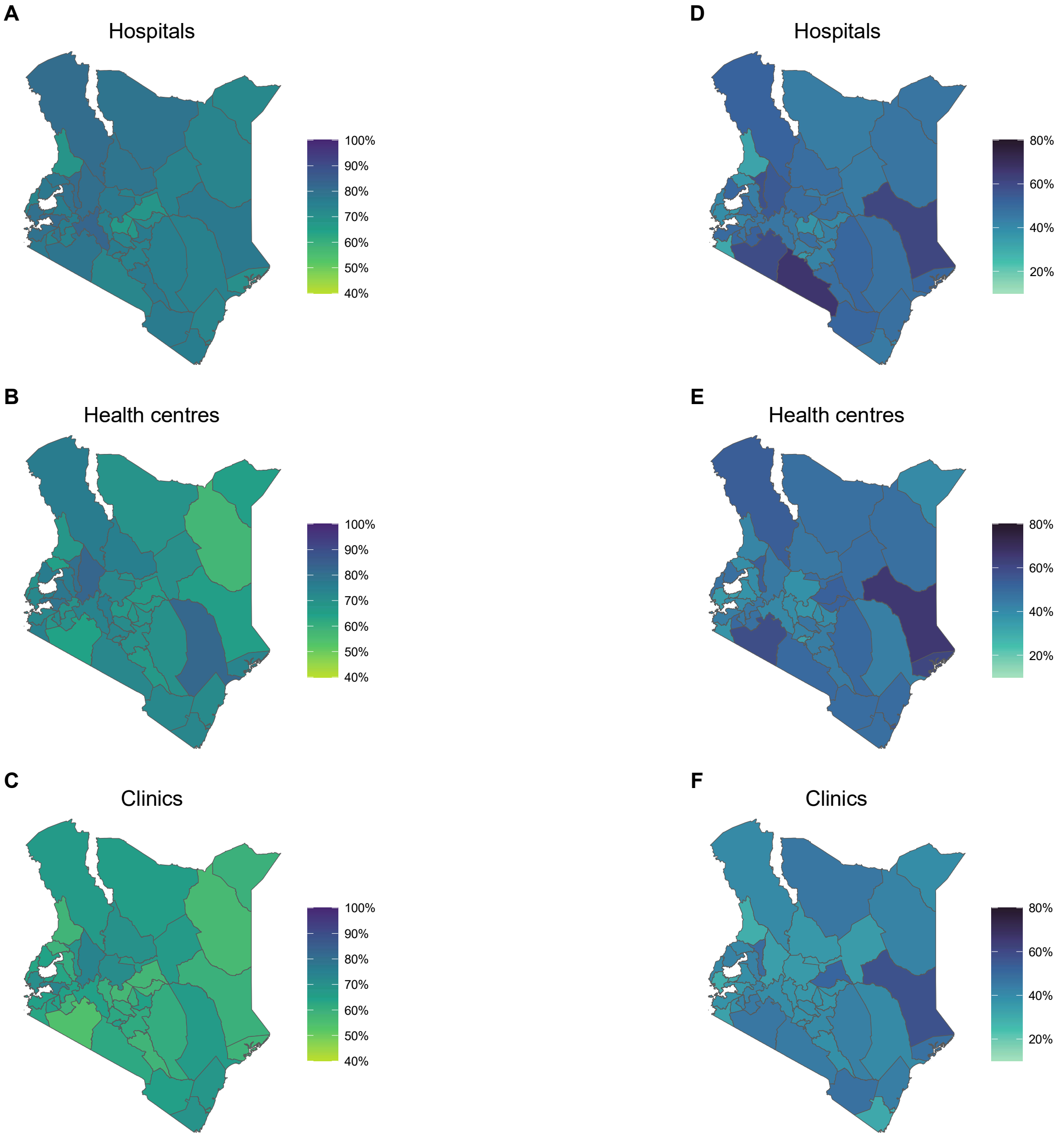

Supplementary Figure S9: Maps of model-estimated readiness (left panel) and process quality (right panel) metrics by subnational areas, and managing authorities, in Kenya in 2020. Figures A, B, and C (respectively D, E, and F) are maps of modelled area-level estimates of readiness (respectively process quality) for analyses stratified on facility type.

Supplementary References

1. Mercer, L. D. *et al.* Space-Time Smoothing of Complex Survey Data: Small Area Estimation for Child Mortality. *Ann. Appl. Stat.* **9**, 1889–1905 (2015).
2. Besag, J., York, J. & Mollié, A. Bayesian image restoration, with two applications in spatial statistics. *Ann. Inst. Stat. Math.* **43**, 1–20 (1991).
3. Simpson, D., Rue, H., Riebler, A., Martins, T. G. & Sørbye, S. H. Penalising Model Component Complexity: A Principled, Practical Approach to Constructing Priors. *Stat. Sci.* **32**, (2017).
4. Rue, H., Martino, S. & Chopin, N. Approximate Bayesian inference for latent Gaussian models by using integrated nested Laplace approximations. *J. R. Stat. Soc. Ser. B Stat. Methodol.* **71**, 319–392 (2009).
5. Lindgren, F. & Rue, H. Bayesian Spatial Modelling with R-INLA. *J. Stat. Softw.* **63**, 1–25 (2015).
6. Faraway, J. J. On the Cost of Data Analysis. *J. Comput. Graph. Stat.* **1**, 213–229 (1992).
7. Spiegelhalter, D. J., Best, N. G., Carlin, B. P. & van der Linde, A. The deviance information criterion: 12 years on. *J. R. Stat. Soc. Ser. B Stat. Methodol.* **76**, 485–493 (2014).
8. Watanabe, S. Asymptotic Equivalence of Bayes Cross Validation and Widely Applicable Information Criterion in Singular Learning Theory. 24.
9. Held, L., Schrödle, B. & Rue, H. Posterior and cross-validatory predictive checks: A comparison of MCMC and INLA. *Stat. Model. Regres. Struct.* 91–110 (2010) doi:10.1007/978-3-7908-2413-1_6.
