# Supplementary material for: A small area model to assess temporal trends and sub-national disparities in healthcare quality
Source: Nat Commun. 2023 Jul 28;14:4555. doi: 10.1038/s41467-023-40234-9 (PMC10382513; doi:10.1038/s41467-023-40234-9)
Supplement: Supplementary file 3 — Reporting summary [file 41467_2023_40234_MOESM3_ESM.pdf]

nature portfolio

Corresponding author(s): Adrien Allorant

Last updated by author(s): Mar 17, 2023

Reporting Summary

Nature Portfolio wishes to improve the reproducibility of the work that we publish. This form provides structure for consistency and transparency in reporting. For further information on Nature Portfolio policies, see our Editorial Policies and the Editorial Policy Checklist.

Statistics

For all statistical analyses, confirm that the following items are present in the figure legend, table legend, main text, or Methods section.

n/a	Confirmed	
☐	☒	The exact sample size (*n*) for each experimental group/condition, given as a discrete number and unit of measurement
☐	☒	A statement on whether measurements were taken from distinct samples or whether the same sample was measured repeatedly
☒	☐	The statistical test(s) used AND whether they are one- or two-sided<br>*Only common tests should be described solely by name; describe more complex techniques in the Methods section.*
☐	☒	A description of all covariates tested
☐	☒	A description of any assumptions or corrections, such as tests of normality and adjustment for multiple comparisons
☐	☒	A full description of the statistical parameters including central tendency (e.g. means) or other basic estimates (e.g. regression coefficient) AND variation (e.g. standard deviation) or associated estimates of uncertainty (e.g. confidence intervals)
☒	☐	For null hypothesis testing, the test statistic (e.g. *F*, *t*, *r*) with confidence intervals, effect sizes, degrees of freedom and *P* value noted<br>*Give P values as exact values whenever suitable.*
☐	☒	For Bayesian analysis, information on the choice of priors and Markov chain Monte Carlo settings
☒	☐	For hierarchical and complex designs, identification of the appropriate level for tests and full reporting of outcomes
☒	☐	Estimates of effect sizes (e.g. Cohen's *d*, Pearson's *r*), indicating how they were calculated

Our web collection on statistics for biologists contains articles on many of the points above.

Software and code

Policy information about availability of computer code

Data collection	No primary data collection was carried out for this analysis
Data analysis	This analysis was carried out using R version 4.1.0. The main statistical model used the R-INLA package version 22.12.16 in R. All code used for these analyses is publicly available online at https://github.com/aallorant/sae_facility_surveys

For manuscripts utilizing custom algorithms or software that are central to the research but not yet described in published literature, software must be made available to editors and reviewers. We strongly encourage code deposition in a community repository (e.g. GitHub). See the Nature Portfolio guidelines for submitting code & software for further information.

Data

Policy information about availability of data

All manuscripts must include a data availability statement. This statement should provide the following information, where applicable:

- Accession codes, unique identifiers, or web links for publicly available datasets
- A description of any restrictions on data availability
- For clinical datasets or third party data, please ensure that the statement adheres to our policy

Input data: The facility-level survey data used in this analysis is publicly available from the DHS (https://dhsprogram.com/) and the World Bank's Service Delivery Indicators (https://www.sdindicators.org/) websites. All covariate data are available from the sources listed in Supplementary Table S 3.
Results: All estimates, including yearly readiness and process quality of care metrics by subnational areas with upper and lower bounds, are available in the GitHub repository described in the "Code availability" section. Data used in the figures of this publication can also be found in the GitHub repository described in the "Code availability."

Human research participants

Policy information about studies involving human research participants and Sex and Gender in Research.

Reporting on sex and gender	NA
Population characteristics	NA
Recruitment	NA
Ethics oversight	NA

Note that full information on the approval of the study protocol must also be provided in the manuscript.

Field-specific reporting

Please select the one below that is the best fit for your research. If you are not sure, read the appropriate sections before making your selection.

☒ Life sciences ☐ Behavioural & social sciences ☐ Ecological, evolutionary & environmental sciences

For a reference copy of the document with all sections, see nature.com/documents/nr-reporting-summary-flat.pdf

Life sciences study design

All studies must disclose on these points even when the disclosure is negative.

Sample size	This observational study incorporated all available survey data sources that met the inclusion criteria as described in the Methods section.
Data exclusions	The 2010 SDI surveys in Senegal and Tanzania and the 2012 SDI survey in Kenya were pilot studies, which only sampled a small number of facilities in selected areas of the countries and were therefore excluded from the main analysis.
Replication	This is an observational study using many years of survey data and could be replicated. This analysis is based on observational data;
Randomization	there no experimental groups.
Blinding	This study uses observational survey data, which did not involve group allocation that would justify blinding.

Reporting for specific materials, systems and methods

We require information from authors about some types of materials, experimental systems and methods used in many studies. Here, indicate whether each material, system or method listed is relevant to your study. If you are not sure if a list item applies to your research, read the appropriate section before selecting a response.

Materials & experimental systems

n/a	Involved in the study	
☒	☐	Antibodies
☒	☐	Eukaryotic cell lines
☒	☐	Palaeontology and archaeology
☒	☐	Animals and other organisms
☒	☐	Clinical data
☒	☐	Dual use research of concern

Methods

n/a	Involved in the study	
☒	☐	ChIP-seq
☒	☐	Flow cytometry
☒	☐	MRI-based neuroimaging
